# A new era in cytomegalovirus vaccinology: considerations for rational design of next-generation vaccines to prevent congenital cytomegalovirus infection

**DOI:** 10.1038/s41541-018-0074-4

**Published:** 2018-09-20

**Authors:** Cody S. Nelson, Betsy C. Herold, Sallie R. Permar

**Affiliations:** 1 Human Vaccine Institute, Duke University Medical Center, Durham, NC USA; 2 Department of Pediatrics, Albert Einstein College of Medicine, Bronx, NY USA

## Abstract

Human cytomegalovirus (HCMV), a member of the beta-herpesvirus family, is the most common cause of congenital infection worldwide as well as an important cause of morbidity in transplant recipients and immunosuppressed individuals. An estimated 1 in 150 infants are infected with HCMV at birth, which can result in lifelong, debilitating neurologic sequelae including microcephaly, sensorineural hearing loss, and cognitive impairment. Natural maternal immunity to HCMV decreases the frequency of reinfection and reduces risk of congenital transmission but does not completely protect against neonatal disease. Thus, a vaccine to reduce the incidence and severity of infant infection is a public health priority. A variety of candidate HCMV vaccine approaches have been tried previously, including live-attenuated viruses, glycoprotein subunit formulations, viral vectors, and single/bivalent DNA plasmids, but all have failed to reach target endpoints in clinical trials. Nevertheless, there is a great deal to be learned from the successes and failures of the HCMV vaccine field (both congenital and transplant-associated), as well as from vaccine development efforts for other herpesvirus pathogens including herpes simplex virus 1 and 2, varicella zoster virus, and Epstein–Barr virus. Here, we review those successes and failures, evaluating recent cutting-edge discoveries that have shaped the HCMV vaccine field and identifying topics of critical importance for future investigation. These considerations will inform rational design and evaluation of next-generation vaccines to prevent HCMV-associated congenital infection and disease.

## Clinical endpoint of congenital HCMV vaccination trials

Over the past 50 years of vaccine development to prevent congenital CMV (cCMV) infection, the field has struggled with how best to clinically evaluate vaccine efficacy. Given that cCMV is somewhat rare at a population-level (1 in 150 pregnancies) and occurs in an extraordinarily vulnerable patient population, what is the most appropriate and practical clinical trial endpoint? Should vaccination seek to reduce viral spread from toddlers, prevent infection of the mother, block viral transmission across the placenta, and/or reduce pathogenesis in the infant?^1^ The endpoint utilized in phase 2 efficacy trials of the glycoprotein B (gB) subunit vaccine was a reduction in the rate of maternal HCMV acquisition.^2,3^ As preexisting natural HCMV immunity is not protective against HCMV reinfection or against viral reactivation, there is a certain degree of pessimism among researchers who contend that vaccine-elicited sterilizing maternal immunity is an unrealistic goal.^4^ Yet, it is quite encouraging to future vaccine development efforts that the gB subunit vaccine demonstrated ~ 50% efficacy in preventing HCMV acquisition in women,^2,3^ and reduced viremia in organ transplant recipients.^5^ Potentially, if the primary outcome of gB subunit vaccination studies had been the prevention of fetal infection, the measured vaccine efficacy might have been higher.

Given that sterilizing immunity against HCMV infection may be difficult to achieve, one alternative approach (or proposal) is to prioritize a reduction in the incidence of fetal infection and/or severity of congenital disease as a clinical endpoint.^1^ Importantly, both guinea pig and rhesus macaque challenge models of cCMV transmission have given confidence to the assertion that vaccines can modulate the incidence and severity of congenital infection. In guinea pigs, immunization with a gB subunit vaccine,^6^ live-attenuated vaccine,^7,8^ or LCMV vector^9^ as well as passive infusion of a gH-specific mAb^10^ have been shown to reduce rates of cCMV infection. Furthermore, we have demonstrated that preexisting HCMV-specific antibody can reduce cCMV transmission in a rhesus monkey model.^11^ These findings justify further preclinical and clinical evaluation of vaccine candidates for their ultimate purpose—to prevent congenital infant infection and disease.

## Successes in herpesvirus vaccine development

Over the past several decades, there have been major advances in herpesvirus vaccine development. Because of the challenge of inducing sterilizing immunity against herpesviruses, vaccine efficacy is frequently assessed for both preventative efficacy (prevention of acquisition) and therapeutic efficacy (improvement of disease). The crowning achievement of herpesvirus vaccine research is the development of safe and efficacious varicella zoster virus (VZV) vaccines to both prevent “chickenpox” and provide a therapeutic reduction in symptomatic shingles and/or postherpetic neuralgia (PHN). A live-attenuated virus vaccine, which demonstrated vaccine efficacy for the prevention of chickenpox disease ranging from 70 to 96% depending on preparation,^12^ was initially approved in 1995. Subsequently, the same vaccine, which was ~ 60% effective against zoster/PHN, gained FDA approval for these additional indications.^13^ More recently, a VZV gE subunit vaccine (combined with the adjuvant AS01B) demonstrated a remarkable 97% efficacy at preventing zoster in clinical trial and was approved for that indication.^14^ It is remarkable that the subunit vaccine outperformed the live-attenuated vaccine, although the dosing regimen was different (two doses of subunit versus single dose of live-attenuated vaccine strain) and immunological responses that contribute to protection may be distinct. This gE subunit vaccine boosts both humoral and polyfunctional CD4 + T-cell responses to gE, which is the most abundant viral glycoprotein expressed by VZV-infected cells.^15^ Finally, though less well-known, there have also been successful vaccination-based eradication campaigns for veterinary herpesviruses including bovine herpesvirus 1.^16^ The HCMV vaccine field stands to learn a great deal from these successes—most importantly that such a vaccine is possible.

The HCMV gB subunit vaccine is the most efficacious tested to date, achieving ~ 50% efficacy in preventing primary HCMV infection in multiple clinical trials.^2,3^ Intriguingly, the use of a subunit vaccine platform has met with mixed success for several other herpesvirus pathogens. A subunit vaccine comprised of glycoprotein D from HSV-2 (gD-2) combined with the adjuvant AS04, initially demonstrated 74% efficacy in HSV-1/2-seronegative women with long-term HSV-2-infected partners, but was not effective in HSV-1 seropositive women or in men.^17^ However, in the larger phase 3 “Herpevac” trial field study, which enrolled > 8000 HSV-1/2-seronegative women, the same vaccine was 20% efficacious (95% confidence interval: −29 to 50%) against all genital herpes disease, although 58% (12–80%) efficacy was observed against genital HSV-1 disease. Efficacy against HSV-1 infection (e.g., seroconversion with or without signs of disease) was 35% (13–52%)) and there was no efficacy against HSV-2 infection (−8%; (−59 to 26%)). A subsequent analysis found that gD-2 binding antibodies, but not CD4 + T-cell responses, correlated with HSV-1 (but not HSV-2) protection.^18^ A subunit vaccine platform has also been attempted for Epstein-Barr virus (EBV). In a phase 2 study, the gp350 vaccine for EBV demonstrated 78% efficacy in preventing infectious mononucleosis, though negligible protection against asymptomatic EBV acquisition.^19^ These partial successes clearly indicate that a subunit vaccine platform to prevent herpesvirus pathology is indeed possible. However, clinical trial data indicate that such herpesvirus subunit vaccines may need to be focused on a subset of herpesvirus-related disease processes (zoster, HSV-1 genital disease, mononucleosis, etc), and may not be effective at preventing infection or eliciting “sterilizing” immunity.

## The challenge of superinfection

Given that the HCMV basic reproductive rate (R_0_) is 1.7 (e.g., each person infects 1.7 others on average),^20^ effective immunity in a mere 41% of the population would restrict population-level transmission if this virus behaved like any other pathogen. One of the largest challenges of HCMV vaccine development remains the ability of this “changeling demon”^21^ to superinfect previously exposed individuals, which recent epidemiologic data has suggested can result in a comparable level of infant congenital disease in seropositive and seronegative populations.^22^ Thus, in addition to a focus on vaccine-elicited protective immunity among HCMV-naïve individuals, future studies should investigate whether vaccination can boost natural immunity and enhance protection against superinfection/reactivation and HCMV-related disease.^23^ Preliminary vaccination studies investigating the use of the gB/MF59 subunit vaccine in seropositive individuals indicate that vaccination can indeed improve upon natural immunity, specifically gB-specific CD4 + T-cell responses.^24^ Furthermore, in a cohort of HCMV-seropositive transplant recipients, gB vaccination boosted neutralizing antibody responses targeting the AD-2 neutralizing epitope, and enhanced AD-2 binding was associated with a reduced incidence of HCMV viremia.^25^

## Humoral vs. cellular protective immunity

It is anticipated that any efficacious HCMV vaccine may have to engage multiple branches of the immune system.^23^ Neutralizing antibodies have been frequently associated with protection against cCMV transmission.^26–29^ Furthermore, an apparent reduction in the rate and severity of cCMV infection was observed following passive infusion of HCMV-neutralizing antibodies to infected women,^30,31^ though this protection was not replicated in a recent phase 3, placebo-controlled trial.^32^ In addition to neutralizing antibodies, high-magnitude HCMV-specific CD4 + T-cell responses have been repeatedly correlated with protection against cCMV infection,^33,34^ suggesting that this cell type is likely critical in maturation of the protective antigen-specific immune response.

The debate over antibodies vs. cellular immune responses as correlates of protection is shared by the HSV vaccine field. HSV gD-binding antibodies, which exhibit neutralizing activity in vitro, were associated with modest protection against HSV-1 disease in the Herpevac trial, although the primary endpoint (protection against genital herpes) was not achieved.^18^ Similar to the HCMV gB vaccine trials,^2,3^ it is unclear whether the limited vaccine efficacy was due to: (1) inadequate neutralizing and/or epitope-specific gD antibody responses,^35^ (2) need for a combination of viral antigens, (3) requirement for non-neutralizing antibody functions such as antibody-dependent cellular cytotoxicity (ADCC), and/or (4) failure to elicit potent antigen-specific T-cell immunity. The highly efficacious live-attenuated VZV vaccine elicits both robust and durable humoral immunity^36^ as well as potent CD4 + /CD8 + T-cell responses.^37^ Furthermore, the VZV gE subunit vaccine stimulates high-magnitude gE-specific antibodies and CD4 + , but not CD8 + , T-cell responses.^38^ These data suggest that humoral and cellular immunity may both be necessary for optimal herpesvirus vaccine efficacy.

Our group previously established a nonhuman primate model for cCMV transmission^39^ observing that depletion of CD4 + T cells prior to primary rhesus CMV (RhCMV) infection resulted in universal congenital RhCMV infection and a high frequency of fetal loss. We subsequently used this model to investigate the ability of preexisting neutralizing antibodies to protect against placental CMV transmission and fetal loss following primary maternal infection. Pregnant, RhCMV-seronegative, CD4 + T-cell depleted dams were treated with hyperimmune globulin purified from RhCMV-seropositive monkeys, then inoculated with RhCMV. Intriguingly, the pre-infused antibody provided complete protection against fetal loss, while the most potently neutralizing HIG product blocked placental RhCMV transmission.^11^ These studies indicate that antibody alone can be protective against cCMV transmission and could be a primary target of vaccines to eliminate this disease. We hypothesize that depletion of CD4 + T cells impacts the incidence of congenital RhCMV infection due to the impact on antibody maturation pathways, though we have not excluded any potential protection of cell-mediated immunity in the rhesus monkey model.

## Immunogen selection

HCMV encodes 165 unique proteins,^40^ yet previous HCMV vaccination efforts have focused almost exclusively on a handful of prevalent and immunodominant antigens including common targets of neutralizing antibodies gB, gH, and UL128-131A as well as T-cell epitopes pp65 and IE1.^23^ To elicit potent humoral immunity, gB seems a logical immunogen choice as it is the viral fusogen,^41^ is highly immunogenic, is the target of neutralizing antibodies, and is necessary for entry into all cell types.^42^ gH is also a dominant target of neutralizing antibodies and part of the core viral fusion machinery,^41^ though this neutralization can be strain-specific.^43^ Finally, the UL128/UL130/UL131a proteins, when assembled with the gH/gL heterodimer to form the HCMV pentameric complex (PC), is necessary for entry into epithelial cells and is the target of the most potent neutralizing antibodies.^44–46^ The robust potency of neutralizing antibodies targeting UL128-131A makes the PC an attractive vaccine target, and indeed much of the HCMV vaccine field is now focused on targeting these epitopes.^47^ However, there is no indication that the magnitude of neutralization assessed in epithelial cells in vitro has any relationship with protective efficacy in vivo.^48,49^ Presumably, HCMV traverses the placenta by spreading from cell-to-cell.^50^ However, there is some disagreement regarding whether PC-specific antibodies can block infection of human placental trophoblasts and thereby prevent cCMV transmission. This process may be influenced by gestational age of the placenta: PC mAbs have been shown to inhibit infection of cytotrophoblasts harvested from term placentas,^51^ but not trophoblast progenitor cells isolated during the first trimester of gestation.^52^

For T-cell epitopes, both pp65 and IE1 have classically been identified as dominant T-cell targets that are present in high frequency in HCMV-seropositive individuals.^23^ Yet, of the 165 proteins encoded by HCMV, 151 have been shown to be targeted by CD4 + or CD8 + T cells.^53^ And it should be noted that the immunodominance of pp65 and IE1-specific T cells does not necessarily reflect the functionality of protective responses, as “subdominant” responses have been shown to be equivalently protective in murine transplant models following adoptive transfer^54^ or DNA vaccination.^55^ More recent analysis of the HCMV-specific CD4 + and CD8 + T-cell repertoire elicited by natural infection suggests that gB and the PC are more commonly targeted by CD4 + T cells, IE1 more commonly by CD8 + T cells, and pp65 by both T-cell subsets.^56^

It has long been theorized that an HCMV vaccine might require a multi-antigen approach, incorporating diverse epitopes to optimally engage both humoral and cellular immune factors, thus maximizing the protective immunity elicited. Multi-epitope immune responses can be achieved either through vaccination with a live-attenuated virus or through delivery and/or in vivo expression of a combination of antigens. This approach is not unique to the HCMV vaccine field, as the successful live-attenuated varicella vaccine-elicited responses against many antigens and a diverse array of multivalent vaccine platforms are in development for HSV-1/HSV-2.^57–59^ The most commonly trialed multi-antigen vaccine for HCMV has been the combination of gB and pp65, either as DNA or co-expressed in a viral vector, which appears safe and highly immunogenic^1^ and has demonstrated additive protection in a guinea pig congenital transmission model.^9^ However, ASP0113, a bivalent DNA vaccine encoding pp65 and gB, did not meet its primary or secondary endpoints in a recent Phase 3 clinical trial of HCMV disease in seropositive hematopoietic stem cell transplant recipients.^60^ Multi-antigen vaccines are logistically challenging to develop^61^—particularly, if future vaccine candidates seek to incorporate the HCMV PC, which requires that five proteins be manufactured, delivered, expressed, and stabilized in a conformationally accurate manner.

Next-generation, “plug and play” vaccine platforms such as mRNA are well suited to the rapid development of multivalent vaccines,^62^ and can elicit both robust humoral and T-cell immunity. For example, gB, PC, and pp65-encoding mRNA molecules might easily be combined for use in a single vaccine. The antigenicity of multiple HCMV mRNA constructs has been recently evaluated, both alone and in combination, and observed to elicit both robust neutralizing antibody and T-cell responses in mice and nonhuman primates.^63^ Reassuringly, there was no discernable interference in the neutralizing antibody responses elicited by vaccination with a single immunogen vs. multiple immunogens. However, the authors did observe an important phenomenon of T-cell epitope competition, as mRNA co-expression of PC and pp65 resulted in robust CD4 + and CD8 + T-cell responses against PC epitopes, but negligible responses against pp65 epitopes. Yet this competition was able to be overcome by an initial priming vaccination with pp65 alone, followed by co-administration of a pp65 and PC mRNA vaccine.^63^

## Neutralizing vs. non-neutralizing antibody responses

The elicitation of neutralizing antibodies has been a primary goal of HCMV vaccine research for the past 40 years.^23^ It has been hypothesized that a cCMV vaccine could be efficacious by inducing HCMV-neutralizing antibodies that either: (1) provide sterilizing immunity by inhibiting HCMV acquisition or (2) reduce systemic viral replication, viral seeding of the placenta, and subsequent fetal infection.^1^ Neutralizing antibodies targeting HCMV surface glycoproteins have repeatedly been correlated with reduced incidence of congenital virus transmission after primary^26–28^ and secondary^29^ maternal HCMV infection. Few studies have evaluated any potential role of non-neutralizing antibodies. However, HCMV is a highly cell associated virus^64^ and “immunologically covert” cell-to-cell spread of HCMV is particularly implicated among wild-type clinical strains of HCMV with an intact PC.^49^ It has become increasingly recognized that neutralizing antibodies cannot block cell-to-cell spread of wild-type HCMV.^65^ Thus, non-neutralizing antibodies might provide an additional benefit by inducing complement-mediated virion lysis or by engaging Fc receptors on immune effector cells that mediate ADCC or antibody-dependent cellular phagocytosis (ADCP).^66^

There is accumulating evidence in the HSV vaccine field that non-neutralizing antibodies may play an important role in vaccine-mediated protection, particularly against HSV-2. The magnitude of gD-specific antibodies was correlated with protection against genital HSV-1, but not genital HSV-2 disease in the Herpevac clinical trial^18^ and it is hypothesized that the results may reflect serotype differences in neutralization.^67^ Given that the gD/AS04 subunit vaccine demonstrated no efficacy against HSV-2 in the Phase 3 field study^68^ a variety of vaccines employing multiple antigenic targets (gC, gD, gE in combination) have been tested and have met with success in animal models of HSV pathogenesis.^69–71^ Furthermore, Genocea Biosciences tested the GEN-003 vaccine containing both soluble gD and the ICP4.2 T-cell epitope in phase 2 trials, resulting in a measurable reduction in viral shedding (though comparable to valacyclovir drug therapy).^72^

In contrast, one intriguing new HSV vaccine candidate is an HSV-2 virus deleted for gD.^73^ In the absence of gD, this vaccine (designated ΔgD-2) provided 100% protection against intravaginal (female) and skin (males and females) challenge with clinical isolates of both HSV-1 and HSV-2.^57,74,75^ Passive transfer studies demonstrated that immune sera alone were sufficient to mediate protection. Importantly, vaccine-elicited antibodies were weakly neutralizing but potently activated Fc-gamma receptors (FcγR) to mediate ADCC and ADCP.^57,74,75^ Protection was lost in FcγR or Fc neonatal receptor knockout mice, indicating a central role for non-neutralizing Abs.^57^ The precise mechanisms by which deletion of an immunodominant viral antigen results in a preferential induction of non-neutralizing antibody responses is under investigation. One hypothesis is that the relative balance of neutralizing and non-neutralizing antibody responses may be regulated, in part, by herpesvirus entry mediator (HVEM), a TNF receptor, and a second-signaling molecule on B cells, T cells, and antigen-presenting cells.^76^ gD competitively binds to HVEM and this interaction may promote the induction of neutralizing or inhibit the generation of non-neutralizing antibodies. In the absence of gD, the balance may be shifted. Notably, CMV *UL144* binds BTLA, one of the ligands for HVEM, and could potentially modulate immune responses against CMV through a similar pathway.^77,78^ Although the studies with the HSV deletion vaccine are limited to date to small animal models, the notion that ADCC Abs may be important is consistent with prior clinical studies demonstrating that maternally acquired HSV-specific antibodies that mediate ADCC provided greater protection against viral dissemination.^79,80^ Furthermore, data have arisen suggesting the protective efficacy of non-neutralizing antibody responses against other viral pathogens including HIV/SHIV^81,82^ and influenza,^83^ although a combination of neutralizing and non-neutralizing antibody response may be required for optimal protection. Further investigation of ΔgD-2 and of FcR antibody responses in mediating HSV protection is warranted.

The role of non-neutralizing antibodies in anti-HCMV immunity is gradually being recognized. A recent study examined the ability of neutralizing and non-neutralizing gB-specific antibodies to reduce viral replication in a murine CMV (MCMV) model when given prior to MCMV inoculation (prophylactically) or following MCMV infection (therapeutically).^48^ When given prophylactically, neutralizing antibodies had the greatest therapeutic effect on viral load, though both neutralizing and non-neutralizing antibodies were equally effective in preventing mouse mortality. In addition, we recently identified that non-neutralizing antibodies were likely responsible for the 50% vaccine efficacy observed in an HCMV gB subunit vaccine trial.^3,84^ In previous phase 1 trials, the gB/MF59 vaccine was observed to elicit a high titer of gB-specific antibodies,^85^ though relatively low-level neutralization^86^ that is enhanced in the presence of complement.^87^ Therefore, the induction of neutralizing antibodies has long been thought to be the mechanism of partial vaccine efficacy. However, in a detailed evaluation of the vaccine-elicited responses in a phase 2 trial of postpartum women gB/MF59 vaccinees,^3^ there were no neutralizing responses detectable against heterologous viral strains, suggesting both: (1) a possible difference in the elicitation of neutralizing antibodies in postpartum women compared to healthy, non-recently pregnant individuals and/or (2) that neutralizing antibodies may not have been necessary for the partial vaccine efficacy.^84^

## Immunogen structural design

There have been significant advances made in the structural biology of viral fusion proteins over the past decade. The structural models that have emerged have revolutionized the vaccine immunology field by facilitating structure-based vaccine design.^88^ Immunogen conformation has been recognized as an extraordinarily important consideration for HCMV gB as well as the gH/gL/UL128-131a PC. gB is a class III viral fusogen,^89^ and is thus a metastable protein that facilitates merger of the viral envelope and host cell membrane by a conformational change from pre-fusion and post-fusion states.^90,91^ The post-fusion crystal structure of the protein has been determined,^92,93^ though the pre-fusion form has remained elusive. Following natural infection, some gB-specific antibodies are neutralizing, though the majority are non-neutralizing.^94^ It has been hypothesized that neutralizing antibodies preferentially target epitopes exposed on the pre-fusion form of the protein, and non-neutralizing antibodies those on the post-fusion form.^92^ Indeed, viral metastable expression of both post-fusion and pre-fusion forms of gB on the virion membrane is proposed as a mechanism of HCMV immune evasion, by which the virus elicits a dominant, non-neutralizing antibody response.^92^ Consistent with this hypothesis, we have previously demonstrated that immunization with soluble post-fusion gB elicited low-level binding responses against neutralizing gB epitopes in comparison with natural infection,^84^ suggesting that neutralizing epitopes are not adequately exposed to immune cells when gB is in the post-fusion form.

There is a high degree of conservation between HCMV gB and that of HSV and EBV.^91,92,95^ We have a great deal to learn from other herpesvirus fields as HCMV structural biology has lagged behind. The HSV post-fusion crystal structure has been published for well over a decade,^96^ and now have the first hints of a pre-fusion structure using cryo-electron tomography technology.^97,98^ Furthermore, structure-based mutagenesis techniques have led to the identification of targeted mutations that can prevent the transition from pre-fusion to post-fusion conformation.^99^ A stabilized pre-fusion form of the protein will be an indispensable tool for future study. The HCMV vaccine field should utilize these advances to pursue both a pre-fusion structure and a stabilized pre-fusion gB construct. Moreover, isolation of antibodies specific for this conformation of the protein, would be a tremendous advance for a reverse vaccinology approach to HCMV.

Finally, the structural biology of the gH/gL/UL128-131a PC is of interest in the HCMV vaccine field. Though extremely potent epithelial cell-neutralizing antibodies are directed against UL128, UL130, and UL131 following natural infection,^46^ immunization with recombinant protein subunits or peptides induces far less potently neutralizing antibodies.^100^ This finding suggests that native folding and assembly of the full complex may be critical for optimal neutralizing antibody responses,^23^ and indeed antibodies have been identified that recognize conformational epitopes only formed by the full PC.^46^ The recently deciphered PC crystal structure^101^ bound to neutralizing antibodies has informed the molecular basis of these conformational epitopes. And wherreas the PC can be expressed in soluble form,^102^ it is unknown how stable this complex is in vivo and thus unknown whether PC subunit vaccination could elicit neutralization titers comparable to natural infection. Thus, many researchers have sought to employ epitope expression strategies such as an MVA vector^103^ or mRNA.^63^

## Antigenic diversity and breadth

It has long been recognized that HCMV is polymorphic between hosts.^104–107^ With the application of next-generation sequencing technology to HCMV, it has become increasingly apparent that there is extensive, genome-wide antigenic variability between viral strains and even within a single host.^108–110^ The HCMV vaccine field appears to face a somewhat unique challenge in combating this antigenic diversity; among herpesvirus, HCMV boasts far-and-away the highest level of interstrain diversity.^111^ Indeed, HCMV has an overall mean distance (i.e., number of substitution per base pair) of 0.027,^111^ which places it in the realm of dengue and other RNA viruses.^108^

Thus, it is perhaps no surprise that antigenic differences have been observed to impact neutralizing antibody responses. In a landmark study, neutralization of HCMV viral isolates was assessed by paired human sera (autologous neutralization) and by the sera of other HCMV-seropositive subjects (heterologous neutralization).^112^ Intriguingly, the authors noted that neutralization antibody titers frequently differed between individuals by an order of magnitude or more, and one viral isolate exhibited complete resistance to heterologous sera antibodies. Indeed, studies of the HCMV and MCMV-specific antibody repertoire have confirmed these findings by identifying mAbs directed at gB and gH that exhibit strain-specific neutralization activity.^113,114^ Thus, antigenic variation will likely be a critical consideration for HCMV vaccine design. Yet it is worth noting that neutralizing antibodies targeting certain conserved epitopes such as gB AD-2^94^ or the UL128/UL130/UL131a proteins^115^ of the PC do not appear to be strain-specific and have been observed to neutralize genetically diverse HCMV strains.

We have observed in a cohort of gB/MF59-vaccinated postpartum women from a phase 2 vaccine efficacy study^3^ that neutralizing antibodies were only detectable against a virus containing the vaccine strain of gB (Towne), though not against heterologous strains AD169 and TB40/E.^84^ However, heterologous neutralizing antibodies were measurable in a cohort of phase 1 healthy adult gB/MF59 vaccinees, suggesting that patient population may impact the breadth of neutralizing antibodies elicited by gB/MF59 vaccination.

## Glycan shielding and decoy epitopes

The purpose of viral envelope protein glycosylation remains poorly understood, though has been described to either: (1) enhance viral immune evasion or (2) facilitate critical viral processes including cellular entry and/or protein processing.^116^ Glycan shielding of susceptible epitopes is a strategy known to be employed by HIV-1 and influenza,^116^ and perhaps also by HCMV.^117,118^ The mutation of highly conserved glycan sites in HCMV gN dramatically enhances viral susceptibility to neutralizing antibodies—including nAbs targeting glycoproteins other than gN.^118^ The structures of HCMV gB^92^ and the pentameric complex^101^ primary targets of neutralizing antibodies, are both heavily glycosylated. Of particular note, for both gB and the PC, neutralizing epitopes are enriched with highly conserved N-linked glycan sites,^93,119^ suggesting that glycans might play a role in preventing antigenic recognition of these susceptible sites. HCMV gB in particular has 18 *N*-linked glycan sites, markedly more than the homologous protein in either HSV-1 or EBV. By careful manipulation of patterns of glycan shielding, it is anticipated that HCMV can elicit a predominantly non-neutralizing anti-gB response profile. For example, heavy glycosylation of the AD-4 and AD-5 regions known to be targeted by neutralizing antibodies^94^ yet sparse glycosylation of predominantly non-neutralizing AD-1 might explain why non-neutralizing AD-1-specific antibodies are the most dominant gB-specific antibody response observed following natural infection.^92^ An alternative hypothesis to glycan shielding is that viral envelope protein glycans can interact with inhibitory immune cell receptors, and thus might modulate either MHC presentation or B-cell activation pathways,^120^ though there are no data to support this theory for herpesvirus pathogens. Although the impact of HCMV envelope protein glycosylation on neutralizing antibody recognition of susceptible epitopes is currently theoretical, it will be an important consideration for future vaccine design efforts.

Analogously to glycan shielding, an immune-dominant decoy response elicited upon vaccination can divert and limit functional immunity against a pathogen. This phenomenon has been described for several viruses, including HIV-1 and HSV. In one HIV vaccine study,^121^ 93% of the HIV-specific B-cell response was observed against gp41—a protein subunit located proximally to the virion membrane and most frequently targeted by non-neutralizing antibodies.^122^ Similarly, in the HSV field, gD subunit vaccines have long been the focus of vaccine development owing to the immune-dominance of this antigen following natural infection, yet they have had limited success in clinical trial.^68^ Thus, it was hypothesized that gD-specific antibodies, though potently neutralizing, may be a decoy immune response that can block the development of more potently functional antibodies. Subsequently, it was observed that a gD-deletion live-attenuated vaccine can elicit robust, protective, non-neutralizing antibody responses.^57,74,75^

**Fig. 1:**
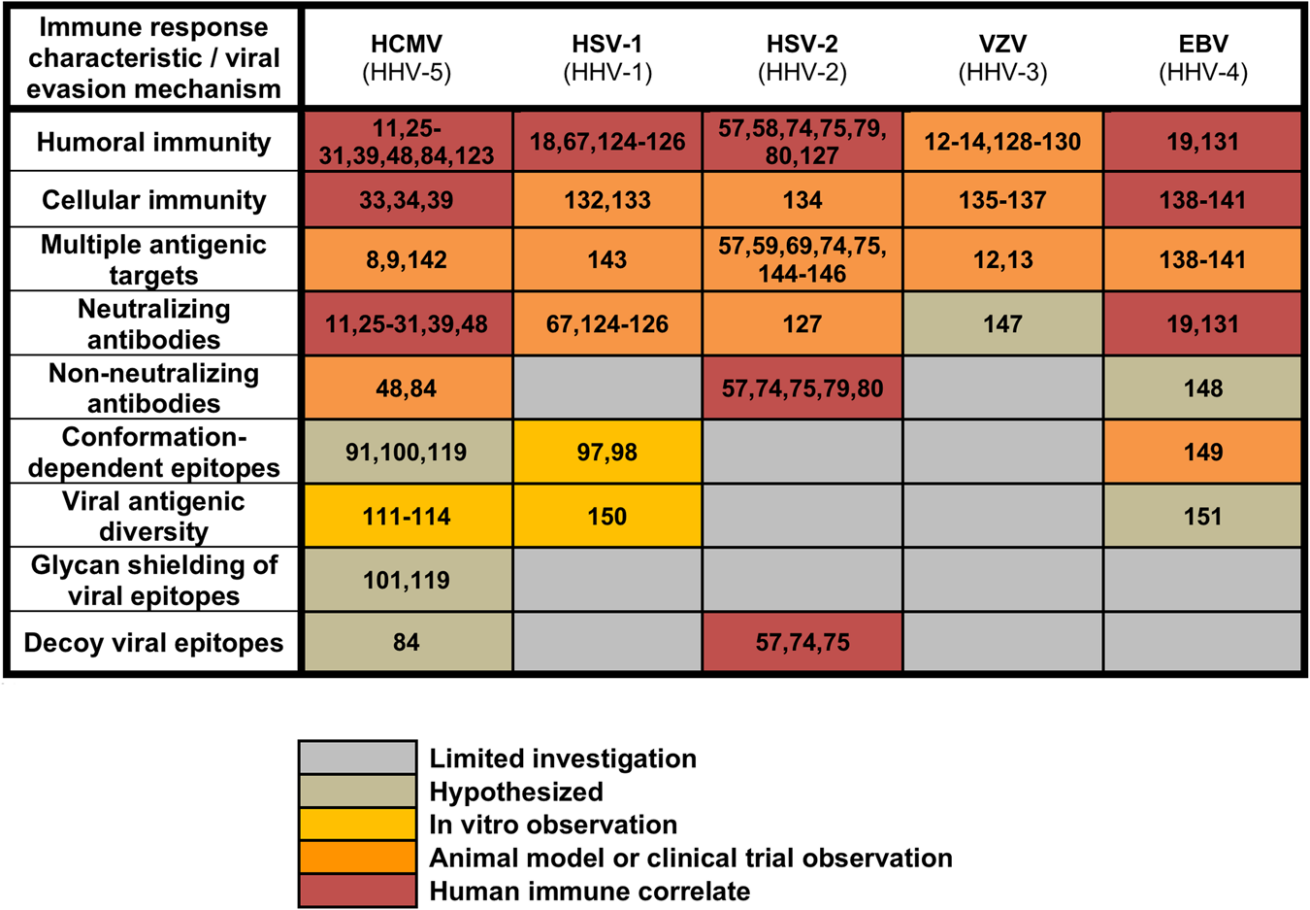
Evidence for virus-specific immune response characteristics and viral immune evasion mechanisms in control of herpesvirus replication and/or prevention of disease. References cited in this figure are:^8,9^^,^^11–14^^,^^18,19^^,^^25–31^^,^^33,34,39,48^^,^^57–59^^,^^67,69,74,75,79,80,84,91,97,98,100,101,111,114,119^^,^^123–151^.

Similarly, we recently assessed the epitope specificity of antibodies elicited by gB/MF59 vaccination in the most efficacious HCMV vaccine trial to date.^84^ Using a peptide microarray covering the full gB open-reading frame, we identified that the gB/MF59 vaccine induced an extraordinarily high-magnitude response against peptide epitopes within the cytosolic gB AD-3 region, a known non-neutralizing epitope.^92^ Indeed, 76% of vaccine-elicited linear gB-binding was directed against the AD-3 epitope, in comparison to 31% in naturally HCMV-infected individuals. As we also observed that this vaccine elicited: (1) very low neutralizing antibody responses and (2) poor targeting of known neutralizing epitopes, we hypothesize that the dominant AD-3-specific antibody response may have diverted functional antibodies away from neutralization-susceptible sites (Fig. 1).

## Final summary

There is an accumulating body of evidence that HCMV vaccination can influence the incidence of infection and congenital disease. There are still numerous questions to be answered and hurdles to be to overcome in HCMV vaccine development; yet interest in the field among both public and private sector researchers has blossomed over the past decade, as evidenced by the number vaccine candidates entering clinical evaluation.^1^ Thus, the future of the HCMV vaccine field is bright, with hope for unique vaccine designs that will bring an end to this common and devastating disease of pediatric and immune-suppressed populations.^123–151^
